# Factors determining diversity of saproxylic arthropods in the fruiting bodies of the birch polypore fungus (*Fomitopsis betulina*)

**DOI:** 10.1186/s13104-025-07434-6

**Published:** 2025-08-18

**Authors:** Craig D. Perl

**Affiliations:** 1 Institute of Aquaculture, University of Stirling, Stirling, FK9 4LA UK; 2 Department of Biology, Indiana University Indianapolis, Indianapolis, IN 46202 USA; 3 Centre for Crop and Environmental Science, Agriculture and Environment Department, Harper Adams University, Newport, Shropshire, UK

**Keywords:** Bracket fungi, Polypore, Saproxylic, Biodiversity, Forest, Biological islands, Sporocarp, Insect, Arthropod

## Abstract

**Objective:**

Certain basidiomycete fungi produce semi-stable fruiting bodies, known as brackets, that support a significant quality of saproxylic invertebrate biodiversity, especially in northern forests. The objective of this investigation was to assess the drivers of saproxylic diversity within the fruiting bodies of *Fomitopsis betulina.* We explore which factors are significant determinants of arthropod diversity and abundance, examining the effects of sporocarp size, height above the ground, and relative isolation from neighbouring sporocarps.

**Results:**

We find that larger sporocarps support a greater number of arthropods, but diversity and species richness are determined by distance from nearest neighbour.

**Supplementary Information:**

The online version contains supplementary material available at 10.1186/s13104-025-07434-6.

## Introduction

Fruiting bodies of some basidiomycete fungi, colloquially called bracket fungi, function as important microhabitats for saproxylic insects and other arthropods, which utilise these sporocarps as food sources, hunting grounds and for physical shelter [1, 2]. The importance of bracket fungi fruiting bodies for saproxylic arthropods [3] has driven investigation into the factors that might influence diversity and distribution among sporocarps. There is evidence that fruiting bodies can act as biological islands (or island-like systems [4]) for invertebrates [5–7], with sporocarp size [6, 7] and nearest neighbour distance found to affect colonisation, abundance and diversity [8].

Alongside sporocarp size and nearest neighbour distance, other variables such as microclimate [7], fungal species [1, 9] and degree of sporocarp decomposition [10] have been explored as determinates of saproxylic species distribution and richness. Bracket microclimate, also thought an important determinant of saproxylic diversity, has been found to be affected by height above the ground [6, 11]. Fruit body size, thickness, surface area, morphology and toughness have all been identified as being drivers of arthropod community structure [12]. Though brackets provide resources to invertebrates independent of their species (such as physical shelter), it has been demonstrated that phylogenetically and ecologically associated brackets on the same tree or log support different invertebrate communities [1, 9], indicating that there is a degree of specificity to interactions, which may be mediated by friability or toughness [12–14] and endogenous biochemistry [11].

The wide variety of factors reported as influential on arthropod diversity in bracket fungi indicates that different factors may be important in a species dependent manner. We used the fruiting bodies of *Fomitopsis betulina*, to generate pilot data on which factors may contribute to arthropod diversity within this system. This species was selected due to its prevalence; with the expectation that a common sporocarp may be exploited by a wide range of arthropods. We found that arthropod abundance increases only with bracket size, and diversity and species richness decrease with increased fruiting body isolation.

## Materials and methods

### Sporocarp collection

**Fig. 1:**
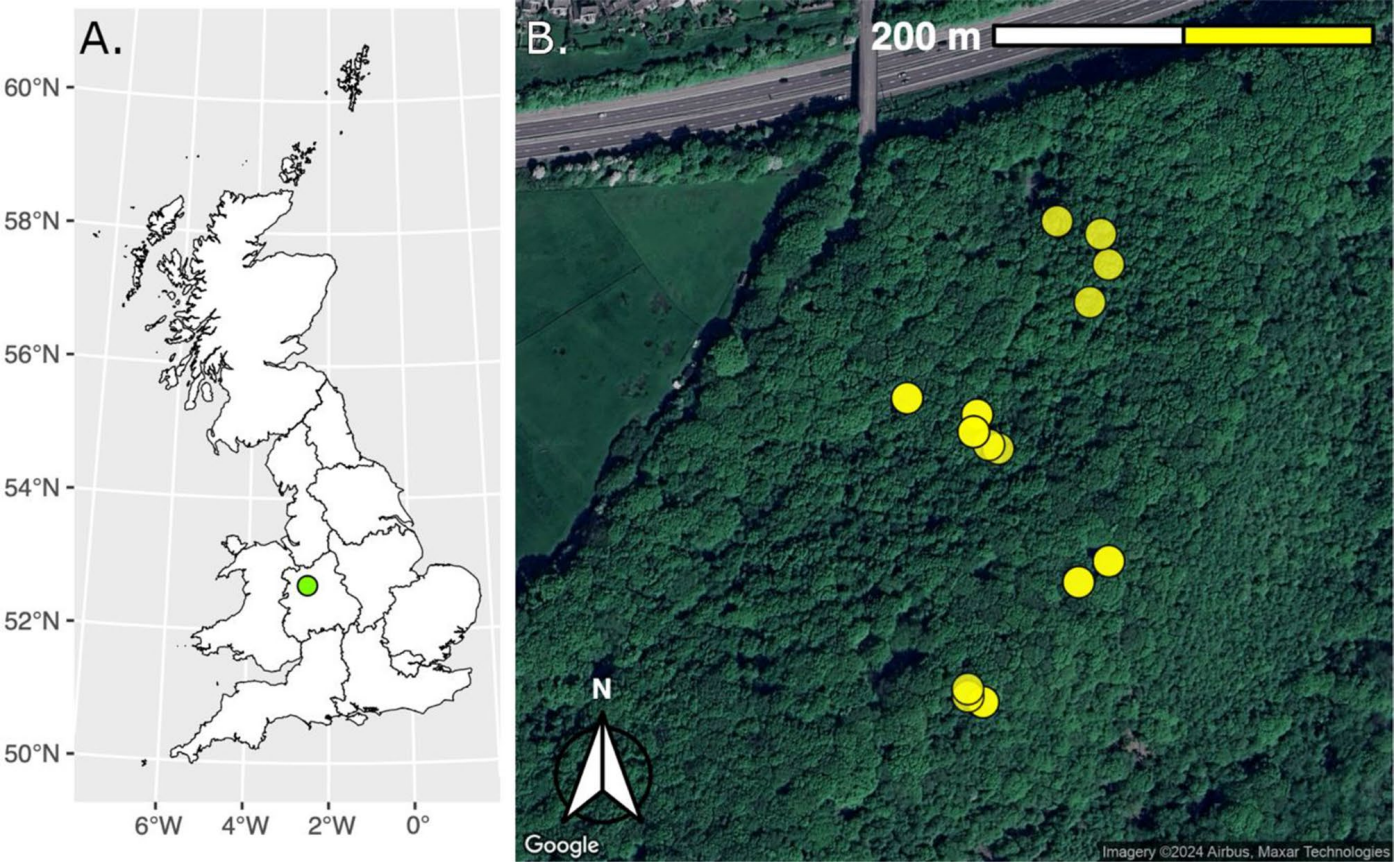
Field site location. **A**: Ercall Wood, Shropshire in the west of England, approximately Ercall Wood is a steep, mixed broad-leaf forest with a maximum elevation of 265 m located at 52°41’18"N, 2°31’19"W. **B**: Location of deadwood from which sporocarps were collected. Each point (of which there are 14) represents a single instance of deadwood from which fruiting bodies were collected. Multiple sporocarps were collected from some dead wood locations.

Eighteen sporocarps from fourteen woodpiles were collected from Ercall Wood, Shropshire, UK 2013/06/16 using random stratified sampling, ensuring collection of sporocarps of a range of sizes. Ercall Wood is a steep, mixed broad-leaf forest with a maximum elevation of 265 m (Fig. 1).

To avoid confounds from decomposition stage; only sporocarps that were over one year old and less than two years old (i.e. sporocarps from the previous year’s growing season) were collected. The collected sporocarps correspond to between stages 2 and 3 in [15] and stage 3 in Graves’ classification [16].

On collection, the height of the sporocarp above the ground, measured as the distance between the soil surface and the lowest point of the sporocarp, was recorded to the nearest centimetre. The number of brackets present on the dead wood were also documented, as was the location of the next nearest *F. betulina* bracket on a different log, ensuring that these nearest neighbours were also between one and two years old. Minimum and maximum distances between fruiting bodies can be found with the raw data and R scripts at 10.17605/OSF.IO/VEWJ8. Only fruiting bodies GPS coordinates were used to establish nearest neighbour distances. The haversine formula tool [17] provided a straight-line distance between two GPS coordinates to the nearest 1 cm. GPS coordinates were recorded using “GPS Location” application (cop-apps.net) installed on a Samsung SIII-mini smartphone. Brackets were collected by hand, wrapped in newspaper and stored in re-sealable plastic bags.

### Sporocarp volume

To measure volume, each sporocarp was placed into a re-sealable plastic sandwich bag from which all the air was expelled. Depending on the size of the bracket, the sporocarp was submerged into a bucket, a 500 ml beaker or a glass tumbler. Each container was filled to the brim with water to ensure maximum displacement. The total volume displaced by the sporocarp was caught in a tray and then measured to the nearest 5 ml.

### Invertebrate identification

Sporocarps were dissected using a scalpel under dissecting binocular microscope. Invertebrates were extracted using fine forceps and a paintbrush before being sacrificed in and stored in 80% ethanol before being sorted into morphospecies (Table S1).

### Diversity

Arthropod abundance, species richness and diversity were recorded from each individual bracket. Diversity was calculated using the Shannon-Wiener index (H’):

$$\:{H}^{{\prime\:}}=-{\sum\:}_{i=1}^{R}{p}_{i}ln{p}_{i}$$

Where *p*_i_ = the proportion of the total individuals belonging to the *i*th species. Analysis was conducted on the effective number of species being a more accurate measure of true diversity and permitting comparisons across diversity indices [18]:

$$\:Effective\:number\:of\:species=exp\left({H}^{{\prime\:}}\right)$$

### Statistics

All statistics were conducted using R v.4.4.0 [19]. Maximal models were initially fitted followed by step-wise elimination of non-significant terms to arrive at the minimum adequate model. Generalised linear models (GLM) and model simplification were all conducted using the R base package. Maximal models were fitted using the following explanatory variables with no interaction terms; log sporocarp volume; nearest sporocarp neighbour; number of other sporocarps at the same log pile; sporocarp height above the ground. Model term significance was assessed using an F-test implemented via the function ‘drop1’, non-significant model terms were eliminated until a minimum adequate model was obtained. Abundance and species richness were overdispersed count data and therefore modelled using a quasipoisson distribution. Effective number of species presented non-normally distributed residuals and were therefore modelled using a gamma distribution.

## Results and discussion

**Fig. 2:**
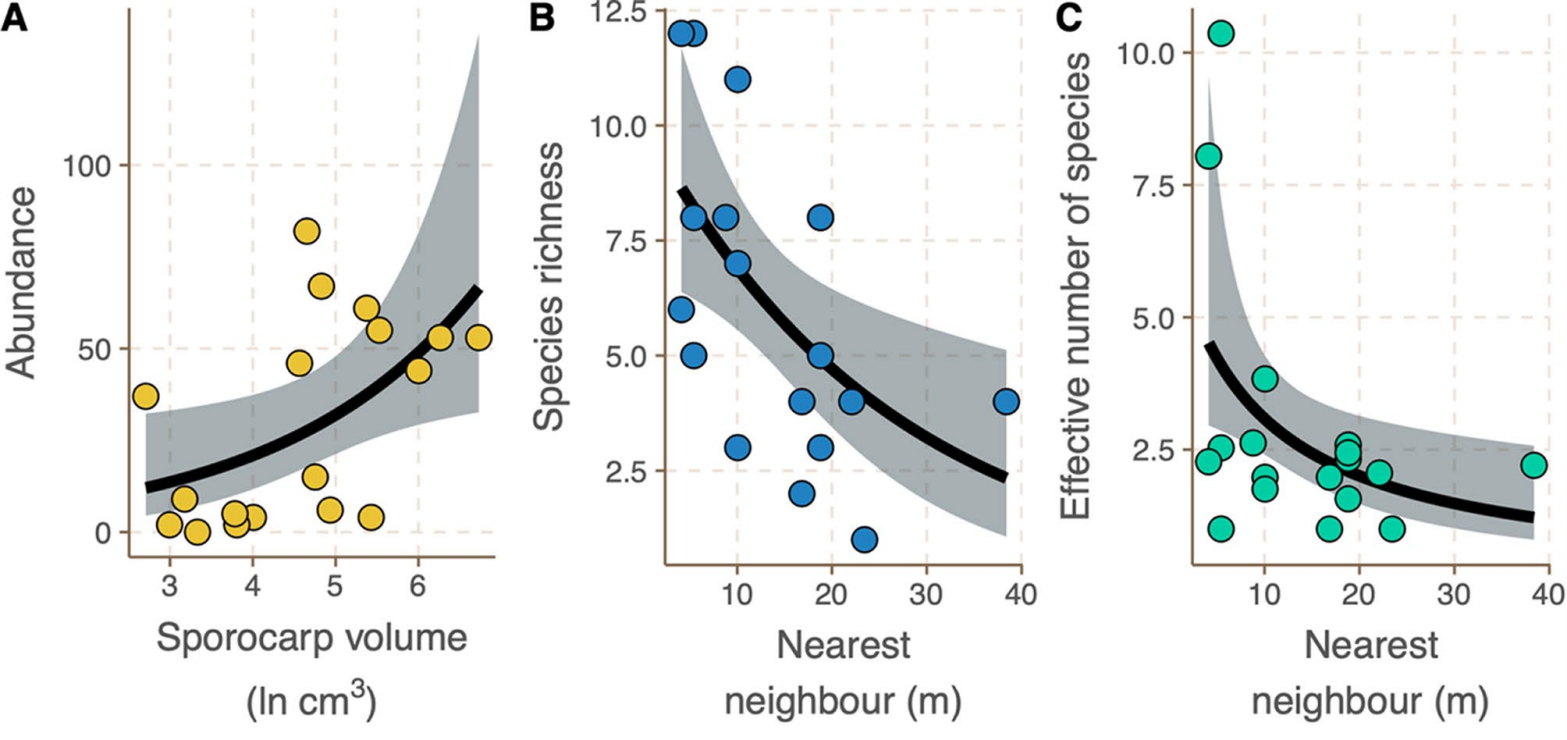
Arthropod abundance, species richness and diversity within *Fomitopsis betulinua* sporocarps. Plots represent minimum adequate models with associated trend lines derived from GLMs. **A**: Arthropod abundance increases with increasing sporocarp volume. **B**: Species richness decreases within a sporocarp with increasing distance from the next nearest sporocarp. **C**: Effective number of species within a sporocarp decreases with increasing distance from the next nearest sporocarp.

545 individuals were sampled, representing 32 morphospecies. There were 6 orders of insect (4 different families of Coleoptera, 3 morphospecies of Diptera, 1 Lepidopteran, 1 Hymenopteran, 1 Hemipteran, 1 Psocopteran), 7 morphospecies of Acari, 2 morphospecies of Araneae, and 2 morphospecies of Diplopoda. We found that sporocarp size was a significant predictor of arthropod abundance (Fig. 2A; GLM, F_1,18_ = 5.29, *p* = 0.035), larger sporocarps supported a greater number of individuals, which is in concordance with other investigations into saproxylic biodiversity [6, 7]. Only distance to the nearest sporocarp neighbour was a significant predictor of species richness (Fig. 2B; GLM, F_1,18_ = 7.70, *p* = 0.014) and arthropod diversity (Fig. 2C; GLM, F_1,18_ = 7.70, *p* = 0.014), both richness and diversity decreased with increasing distance to nearest neighbour [11]. The height of the sporocarp above the soil and the number of sporocarps present on the same piece of dead wood had no significant effect on arthropod abundance, species richness or diversity (GLM, F_1,18_ < 2.40, *p* > 0.14).

These results in part support the concept that bracket fungi sporocarps operate as biological islands; where species richness and diversity may decrease with increased distance to their nearest neighbour. Distance from nearest neighbour may be less relevant to winged groups that can disperse distances greater than the study area. Other groups (e.g. Carabidae) are not mycophagous and may be utilising sporocarps for hunting or for physical shelter. For those species that do not directly rely on sporocarps as a resource, biological island theory would not apply as sporocarps would not act as islands for those individuals. Island biogeography theory also predicts that larger ‘islands’ will support greater diversity, in line with general species-area affects; increased area (or volume, in this instance) is predicted to support greater diversity by providing a wider range of ecological niches that can be exploited [20]. We do not observe this pattern in these data, with increased sporocarp volume supporting greater abundance but having no effect on effective number of species (an indicator of diversity), contrasting with other investigations [5–7]. Our data may differ from previous results due to differences in focal fungal species and the size therein of the fruiting bodies or due to differences in saproxylic species (e.g. size-diversity relationships may only apply to Coleoptera [6, 7]. The limited geographic scope of the survey may also contribute to these observed differences. Other studies found that height above the soil, an indicator of microclimate [7], was an important determinant of diversity, a factor not significant in our analysis.

### Limitations

The most significant limitation to this work is the small number of *F. betulina* fruiting bodies that were collected. To draw stronger conclusions regarding the data collected, a much greater sample size, preferably sampling over a wider geographical area would be needed. Additionally, sampling over a longer period would provide a better indication of the variability of saproxylic species utilising these sporocarps, as would rearing insects from the fruiting bodies rather than dissecting them. This is especially important for identifying any juvenile stages of parasitoids, a group which can be especially speciose. The lack of genus or species level identification, rather than morphospecies, is also a limitation on these results, making it difficult to ascertain which individuals are utilising which sporocarp features.

## Supplementary Information

Below is the link to the electronic supplementary material.

Supplementary Material 1

## Data Availability

Data are available via The Open Science Framework: 10.17605/OSF.IO/VEWJ8.
